# Genome-wide simple sequence repeats (SSR) markers discovered from whole-genome sequence comparisons of multiple spinach accessions

**DOI:** 10.1038/s41598-021-89473-0

**Published:** 2021-05-11

**Authors:** Gehendra Bhattarai, Ainong Shi, Devi R. Kandel, Nora Solís-Gracia, Jorge Alberto da Silva, Carlos A. Avila

**Affiliations:** 1 Department of Horticulture, University of Arkansas, Fayetteville, AR 72701 USA; 2 Texas A&M AgriLife Research and Extension Center, Weslaco, TX 78596 USA; 3 Department of Crop and Soil Sciences, Texas A&M University, College Station, TX 77843 USA; 4 Department of Horticultural Sciences, Texas A&M University, College Station, TX 77843 USA

**Keywords:** Genetic markers, Genotype, Plant breeding, Plant genetics, Population genetics

## Abstract

The availability of well-assembled genome sequences and reduced sequencing costs have enabled the resequencing of many additional accessions in several crops, thus facilitating the rapid discovery and development of simple sequence repeat (SSR) markers. Although the genome sequence of inbred spinach line Sp75 is available, previous efforts have resulted in a limited number of useful SSR markers. Identification of additional polymorphic SSR markers will support genetics and breeding research in spinach. This study aimed to use the available genomic resources to mine and catalog a large number of polymorphic SSR markers. A search for SSR loci on six chromosome sequences of spinach line Sp75 using GMATA identified a total of 42,155 loci with repeat motifs of two to six nucleotides in the Sp75 reference genome. Whole-genome sequences (30x) of additional 21 accessions were aligned against the chromosome sequences of the reference genome and in silico genotyped using the HipSTR program by comparing and counting repeat numbers variation across the SSR loci among the accessions. The HipSTR program generated SSR genotype data were filtered for monomorphic and high missing loci, and a final set of the 5986 polymorphic SSR loci were identified. The polymorphic SSR loci were present at a density of 12.9 SSRs/Mb and were physically mapped. Out of 36 randomly selected SSR loci for validation, two failed to amplify, while the remaining were all polymorphic in a set of 48 spinach accessions from 34 countries. Genetic diversity analysis performed using the SSRs allele score data on the 48 spinach accessions showed three main population groups. This strategy to mine and develop polymorphic SSR markers by a comparative analysis of the genome sequences of multiple accessions and computational genotyping of the candidate SSR loci eliminates the need for laborious experimental screening. Our approach increased the efficiency of discovering a large set of novel polymorphic SSR markers, as demonstrated in this report.

## Introduction

Cultivated spinach (*Spinacia oleracea* L.) is an important cool-season leafy vegetable crop. Spinach is dioecious, wind-pollinated, and a highly heterozygous species. From the nutrition perspective, spinach is an excellent source of health-promoting compounds and nutrients^1^. Spinach is a nutrient-dense food that contains high amounts of vitamins, proteins, minerals, flavonoids, and antioxidants but low in calories^2,3^. The demand for fresh market spinach has doubled in the last decade^4^, probably because of the health benefit and year-round availability of cleaned packaged spinach. The United States annually produces 0.43 million tonnes of spinach with a product value of 425 million dollars^4^, and organic production comprises nearly 50% of the total production in the United States.

Simple sequence repeats (SSR), also known as microsatellites, are DNA segments with a tandem repeat motif of 1–6 nucleotides. Genome-wide coverage, robust and high reproducibility, co-dominant inheritance, high polymorphism with multiple alleles per locus, transferability between species, and low requirements of expertise and instrumentation are some of the attractive features of SSR markers. Hence, SSR markers are relatively low cost for genotyping plants and can be used by small labs. SSR markers have been applied in fingerprinting, genetic diversity studies, population structure analysis, association mapping, and linkage mapping^5–8^. All such applications support to increase fundamental genetics research and plant breeding activities. SSR markers are being developed in many crops^9–12^ and plant pathogens^13–15^ in recent years.

In spinach, a limited number of useful SSRs have been reported. In a small panel of 33 hybrid cultivars using 13 SSR markers, genetic diversity studies found clustering of cultivars into three genetic groups based on origin from different breeding stations^16^. Another study used a set of six SSR markers to investigate genetic diversity in 50 spinach accessions and showed the spinach accessions had high genetic diversity and reported that the accessions from West Asia (Afghanistan, Iran, Iraq, and Syria) had the highest genetic diversity^17^. Furthermore, a search on the bacterial artificial chromosome (BAC) end sequences identified 100 SSR markers showing multiple PCR bands, but the markers were not used to fingerprint or assess diversity^18^. Another study reported a set of 85 polymorphic SSR markers mined from genome sequences of a spinach cultivar, and the loci were genotyped in a set of 48 worldwide spinach accessions and found clustering of spinach accessions based on geographical origin^19^. Recently, 34 new polymorphic SSR markers were developed from the spinach reference genome of Xu et al.^27^ and were used to assess the genetic diversity of Chinese spinach germplasm collection^20^. A fewer number of SSR markers have been reported in spinach; thus, the availability of a large number of useful SSR markers will facilitate spinach genetics and breeding.

The origin of spinach is believed to be in the former Persia; however, domestication and migration have not been fully elucidated^21^. The *Spinacia* genus contains two wild species: *S. turkestanica* and *S. tetrandra*. Both the wild *Spinacia* species are native to Central Asia in the Caspian sea area over the two major geographical regions. The *S. turkestanica* is native to Central and South Asia in Turkmenistan, Uzbekistan, Kazakhstan, Tajikistan, Afghanistan, and Pakistan, while the *S. tetrandra* is native to Transcaucasia and Kurdistan region in Armenia, Georgia, Iran, Iraq, and Turkey^22–24^. Spinach is believed to have migrated to China via Nepal during the seventh century, and later to Europe and very recently to the United States^25^. Recent studies were conducted to explore the genetic diversity of spinach germplasm, including the wild *Spinacia* species, and explore crop ancestry and domestication region that support migration of spinach to China via Nepal^26^. Both wild species are interfertile with the cultivated *S. oleracea* suggesting close genetic relation and recent domestication history^26^. The genetic evidence suggests that *S. turkestanica* is the progenitor of cultivated spinach^26,27^. There is a great opportunity to explore the genetic diversity of *Spinacia* accessions to use them in spinach improvement and by using molecular markers to perform fingerprinting, genetic characterization, marker-trait association, and to incorporate the new findings into molecular breeding programs for cultivar development.

The development of new SSR markers used to be an expensive and time-consuming effort until recently. With the rapid development of sequencing technologies and reduced sequencing cost, genome assemblies are now available for many crops facilitating the rapid and efficient development of SSR markers^12,28^. The resequencing of multiple accessions is common for many commercially important crops with the advancements in sequencing technology. The reference genome assemblies and resequencing reads can be efficiently used to identify and profile a large number of genome-wide polymorphic SSR markers. Several recent bioinformatics tools such as LobSTR^29^, RepeatSeq^30^, STRViper^31^, and HipSTR^32^ have adopted an improved method to identify and genotype SSR alleles using the reference assembly and a set of whole-genome sequence data from multiple samples/individuals.

The genome sequence of inbred spinach line Sp75 is available^27^; however, a limited number of SSR markers have been reported in spinach. In addition, whole-genome resequencing (30x) of 21 spinach accessions were recently generated by our group. This study aimed to explore the chromosome sequences of the Sp75 genome to identify SSR-containing regions and their genomic distribution, identify a larger set of genome-wide polymorphic SSR markers via in silico genotyping using the genome sequences of multiple spinach accessions, and validate a subset of markers following PCR amplification and fragment sizing. The present study is the first comprehensive report of mining and cataloging several thousand polymorphic SSR markers with a known physical position in the spinach genome. The potential of in silico identified polymorphism was further validated following a standard molecular assay, and the usefulness of the markers was confirmed by conducting genetic diversity assessment of the worldwide germplasm accessions. Identifying a large set of SSR markers using this approach provides a cost and resource-efficient alternative to the traditional SSR discovery approaches, which involves random testing of primer pairs designed from the genome and transcriptome sequences.

## Materials and methods

### Plant material

A set of 48 diverse spinach accessions comprising USDA and differential accessions were used for molecular validation and diversity analysis in this study (Supplementary Table S1). Seeds were obtained from the USDA-GRIN spinach germplasm collection at the USDA-ARS North Central Regional Plant Introduction Station, Ames, Iowa. The USDA accessions were originally collected from 34 countries representing worldwide diversity, including 36 *S. oleracea* and three *S. turkestanica* accessions. Plants were grown in the greenhouse condition for 2–3 weeks and leaves were collected for DNA extraction.

### SSR mining

**Figure 1:**
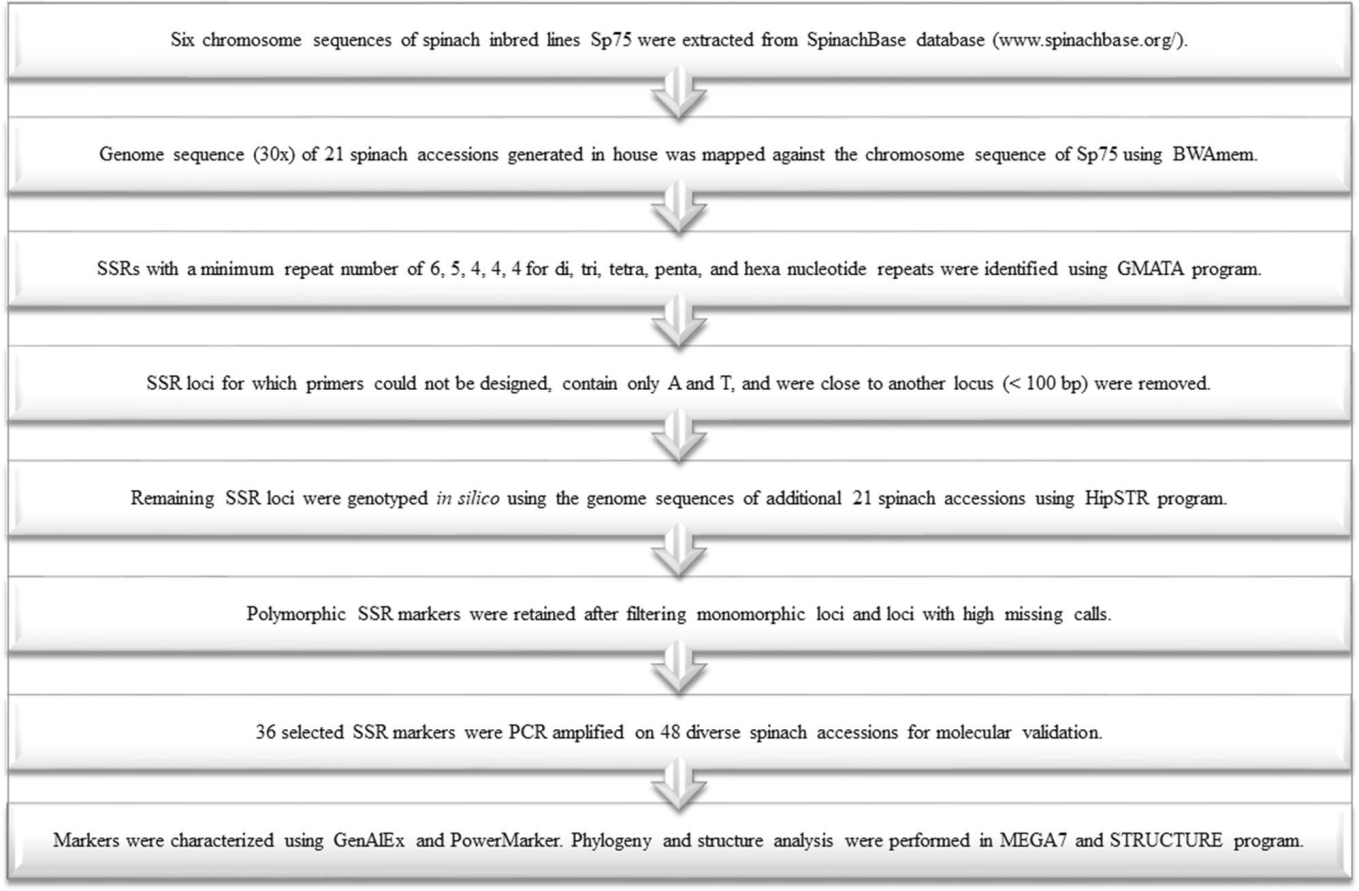
Outline of the approach used to discover SSR loci from the genome sequence, the stepwise procedure employed to identify a large set of polymorphic loci, and genetic characterization and diversity analysis.

The reference genome sequence of spinach inbred line Sp75 is publicly available^27^ in the SpinachBase database (www.spinachbase.org/). The genome was assembled to 996 Mb with six chromosomes and 77,293 scaffolds. The overall procedure used to mine SSRs and identify genome-wide polymorphic SSR loci is outlined in Fig. 1. Briefly, the six spinach chromosome sequences were extracted from the genome assembly and the extracted sequences were searched for SSRs using the GMATA program^33^. The SSR loci were searched for dinucleotide to hexanucleotide repeat motifs with a minimum repeat number of 6, 5, 4, 4, 4 for di, tri, tetra, penta, and hexanucleotides repeats, respectively. Primer pairs were designed using the default parameters in GMATA with the product size ranging from 100 to 400 bp, the annealing temperature of 60 °C (59–61 °C), minimum GC of 40%, and primer length of 20 bp (18–25 bp) using the Primer3 algorithm. Next, the SSR loci for which primers could not be designed were removed and 250 bp flanking sequences on either side of the SSR motifs were extracted for the loci for which primers were designed. Next, the SSR motifs containing only A and T were removed as they are known to be difficult to score^12^, and the SSR loci lying less than 100 nucleotides apart were removed. Finally, the remaining filtered set of SSR loci were pursued to look for polymorphism as described below.

### In silico polymorphism screening

The genome of 21 spinach accessions were resequenced to a depth of 30× using the Illumina NovaSeq machine at Novogene. The genome sequences of the 21 accessions (Califlay, Campania, Clermont, Dolphin, F380, Lazio, Lion, Meerkat, PI4, PI6, PV1202, PV1206, Pigeon, Resistoflay, S08_350, S906304, T14_717, Viroflay, Whale, X10_539, and X97_313) were aligned against the chromosome sequences of the Sp75 assembly to investigate variation in the number of SSR repeat units among the genome sequences of multiple spinach accessions. Initially, the paired-end reads of each of the 21 spinach accessions were mapped to the chromosome sequences of the reference genome using BWA mem v.0.7.17^34^, and SAMtools v.1.9^35^ was used to convert the SAM to BAM format, to discard the unmapped reads, and to sort and index the final alignment file. The mapping and in silico genotyping analysis was performed using the High Performance Computing Center (AHPCC) at the University of Arkansas.

Next, for the filtered set of SSR loci obtained from the GMATA run, a bed file was prepared for each of the selected SSR loci that contain chromosome name, start and end position of SSR loci, motif length, the number of repeat units in the reference sequence, and the SSR locus name. The aligned bam files of the 21 accessions, the bed file containing the SSR regions in the reference genome, and the reference chromosome sequence used to mine SSRs were used to allelotype in the HipSTR program^32^. The HipSTR program was run using the external stutter models and SSR calling with de novo allele generation mode. The HipSTR generated VCF files with SSR calls were filtered for low quality calls using HipSTR option: -min-call-qual 0.9 -max-call-flank-indel 0.15 -max-call-stutter 0.15. Furthermore, the genotype calls were filtered for monomorphic and high missing rates among the accessions and non-reference alleles on less than two accessions. Stepwise removal of monomorphic, high missing, and less interesting loci, a large set in silico genotyped polymorphic loci were retained (Fig. 1). The physical map was drawn in R using the start position of the polymorphic SSR markers, and the markers were colored uniquely for each motif lengths (di- through hexa-). In addition, the genome coordinate of the polymorphic SSRs was compared with the general feature format (GFF) file to identify SSRs distribution within the genic or intergenic regions, and gene function annotations were retrieved from the SpinachBase database.

### SSR markers polymorphism validation

From the set of in silico identified polymorphic SSR loci, 36 loci distributed randomly across all six chromosomes, and all motifs types (di, tri, tetra, penta, and hexanucleotide repeat motifs) were selected for molecular validation. Primer pairs previously designed in GMATA programs were used for PCR. However, for 19 SSR loci, primer pairs were redesigned using Primer 3^36^ to change the expected product size to fit PCR multiplexing and Capillary electrophoresis fragment sizing. All primer pairs were designed with lengths of 20–23 bp, the annealing temperature of 60 °C, and the product size range of 100–400 bp. Also, the 36 primer pairs were designed to fit in six multiplexed sets, with each set consisting of six SSR loci. Primer pairs were then used to genotype 48 USDA spinach accessions and commercial cultivars (Supplementary Table S1). The 48 diverse spinach panels used in the molecular validation also contain nine differential cultivars (Viroflay, Resistoflay, Califlay, Clermont, Lion, Lazio, Whale, Pigeon, and Meerkat) with resequencing data that were initially used to identify polymorphic SSRs following HipSTR analysis. The DNA was extracted using the CTAB buffer (OPS Diagnostics, Lebanon, New Jersey, USA) from three to five young plants per accession.

The PCR reactions were performed as the previous method^37^ using the M13 tailing PCR procedure^38^. The forward primers were tailed by adding an M13 sequence labeled with IRDye to the 5′ end. The PCR reaction was run with the following conditions: 94  °C for 5 min, then 29 cycles at 94  °C for 30 s, 60  °C for 45 s, 72  °C for 45 s, followed by 7 cycles of 94  °C for 30 s, 55  °C for 45 s, 72  °C for 45 s, and a final extension at 72  °C for 10 min. After amplification, 1 µl of each PCR product was mixed with 30 µl of 2X formamide loading buffer (LI-COR, Inc., Lincoln, Nebraska, USA). The resultant mixture was denatured for 5 min at 94  °C and then visualized on 6% denatured polyacrylamide gel using a 4300 DNA analyzer (LI-COR, Inc., Lincoln, Nebraska, USA). Markers were scored as 1 or 0 for the presence and absence of alleles. The binary scores matrix was used for further genetic analysis.

### Genetic diversity, population structure and phylogeny analysis

Amplified loci showing two or more scorable bands among the spinach accessions were used for genetic characterization. Expected heterozygosity (H_e_) or gene diversity was computed in GenAlEx 6.5^39^, while the number of alleles and polymorphism information content (PIC) for the SSRs were calculated using PowerMarker v3.25 software^40^.

Population structure was inferred using Bayesian clustering implemented in STRUCTURE v.2.3.4^41^. Structure analysis was run for K (number of subpopulations) values ranging from 1 to 10 using the admixture ancestry and correlated allele frequency model. Ten independent runs for each K were performed with the burn-in length of 200,000 and MCMC repetitions of 200,000 to estimate the number of the subpopulation. The optimal K was determined using the delta K estimation method^42^ using STRUCTURE Harvester^43^. Spinach accessions were assigned to the subpopulations based on the cluster assignment probability (Q). The cluster assignment probability (Q) value of 0.50 was used to assign spinach accessions to each subpopulation. The bar plot was drawn using the Q matrices for the optimal K in R version 3.6.3 (R Core Team, Vienna, Austria).

The population structure was further confirmed using principal component analysis (PCA) in TASSEL 5.2.65^44^. The PCA plot was drawn in R using the PCA matrices and adding the accessions ID, Q group assignment from STRUCTURE analysis, and species information. Further, phylogenetic trees were drawn using the maximum likelihood method based on the Tamura-Nei model^45^ in MEGA 7^46^ by applying Neighbor-Join and BioNJ algorithms to a matrix of pairwise distances estimated using the Maximum Composite Likelihood (MCL) approach. To ease visualization, STRUCTURE generated Q matrices were imported as the group name in MEGA7, and the branch shape, nodes, and branch line of the phylogenetic tree were drawn with the same color as in the STRUCTURE bar plots and PCA plots.

## Results

### SSR frequency and distribution

**Figure 2:**
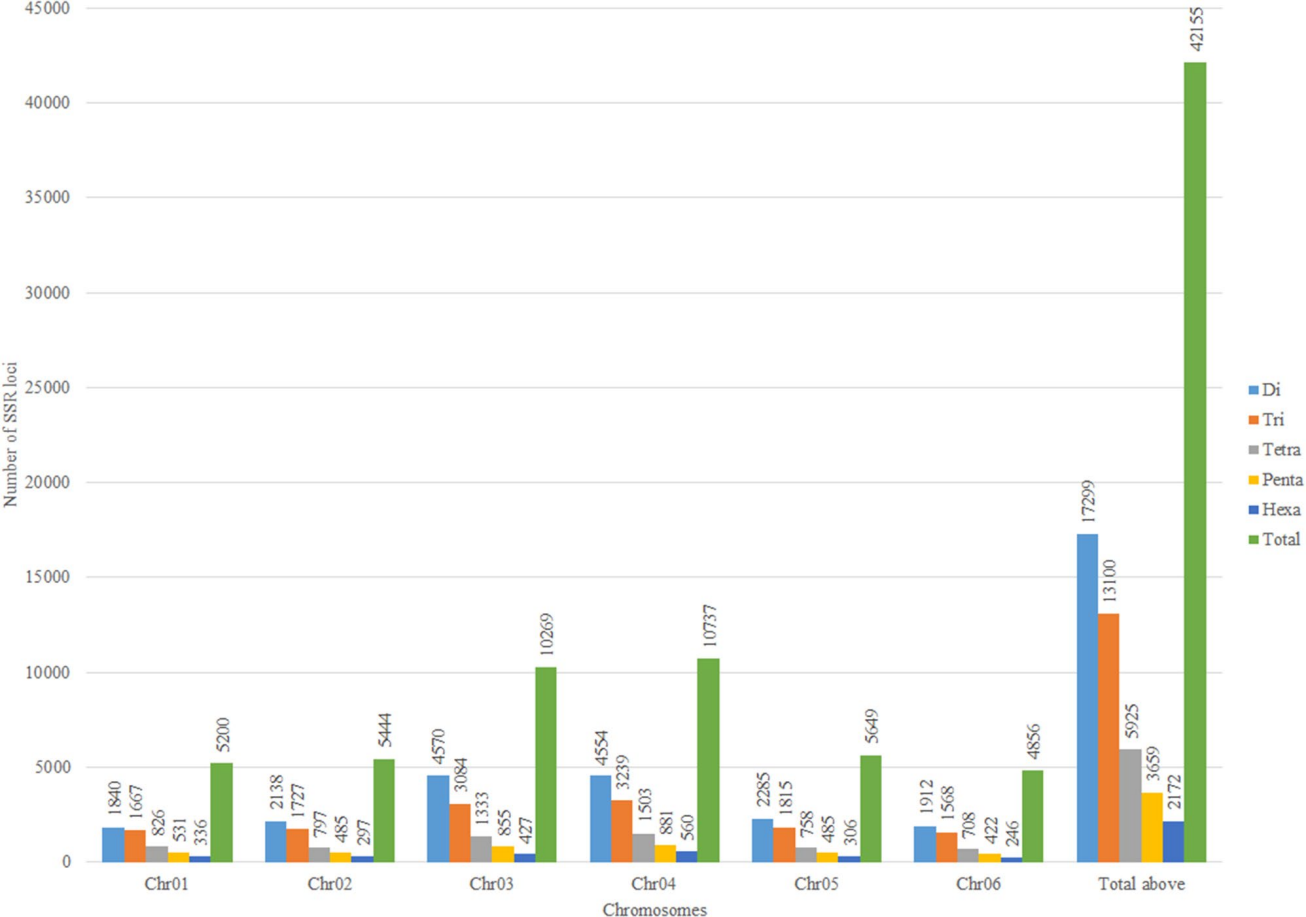
Chromosome-wise distribution of di, tri, tetra, penta, and hexanucleotides repeat SSR loci in the spinach genome.

The reference spinach genome was assembled to 996 Mb^27^ and the chromosome sequence comprised 463.4 Mb, representing about 47% of the genome assembly. The chromosome sequence was searched for dinucleotide to hexanucleotide repeat motifs with a minimum repeat number of 6, 5, 4, 4, 4 for di, tri, tetra, penta, and hexanucleotides repeats, respectively. A total of 42,155 SSR loci (excluding mono-nucleotides) were identified in six spinach chromosome sequences (Tables 1, 2). The SSR loci identified with the given parameters in this study were distributed at a higher density (total SSRs/Mb) on chromosome 6 and chromosome 1 compared to the rest of the chromosomes (Table 1). The frequency of SSRs were 102.6, 89.9, 90.5, 87.3, 81.3, 104.6 loci/Mb across chromosome 1 through 6, respectively (Table 1). The dinucleotide repeat was most abundant, comprising 41.03%, followed by tri, tetra, penta, and hexa repeat SSR comprising 31.07, 14.05, 8.67, 5.15%, respectively (Table 2, Fig. 2).

**Table 1:** Chromosome-wise distribution and density of the simple sequence repeat (SSR) loci in the spinach genome.

Chromosome	Chromosome size (bp)	No. of SSRs	No. of SSRs/Mb	No. of polymorphic SSRs	No. of polymorphic SSRs/Mb
Chr01	50,662,332	5200	102.6	862	17.0
Chr02	60,576,128	5444	89.9	820	13.5
Chr03	113,461,288	10,269	90.5	1277	11.3
Chr04	122,945,393	10,737	87.3	1529	12.4
Chr05	69,506,562	5649	81.3	777	11.2
Chr06	46,407,490	4856	104.6	721	15.5
Total	463,559,193	42,155	–	5986	–
Average	–	–	90.9	–	12.9

**Table 2:** Distribution of simple sequence repeat (SSR) marker in Sp75 genome. Stepwise screening of genome-wide SSR loci to develop polymorphic SSRs is noted. Selected SSR loci were computationally genotyped using the genome sequences of additional accessions. The SSR loci varying in repeating units among the 21 spinach accessions were identified as a polymorphic marker.

Motifs	Total	Primer designed	Distance ≤ 100 bp	Only A&T	HipSTR pursued	HipSTR calls	Mono- and missing at all genotype	No. of loci with alternate allele < 2	Polymorphic loci
Di	17,299	15,459	675	6640	8144	6729	4021	701	2007
Tri	13,100	11,072	391	4847	5834	4963	2328	660	1975
Tetra	5925	4022	40	2349	1633	1427	803	188	436
Penta	3659	2935	445	477	2013	1613	813	198	602
Hexa	2172	2079	53	253	1773	1447	336	145	966
Total	42,155	35,567	1604	14,566	19,397	16,179	8301	1892	5986

### Polymorphic SSRs identification through in silico genome sequence comparison

The SSR loci identified from the GMATA program were investigated in a stepwise manner to identify genome-wide polymorphic loci (Fig. 1, Table 2). Primer pairs were successfully designed for 35,567 of the total SSR loci and were pursued further in the study. Of these, 14,566 SSRs contained only A and T and were removed. Similarly, 1604 SSR loci were near located (less than 100 bp) and removed. The remaining 19,397 SSR loci were pursued to investigate polymorphism using the HipSTR program (Table 2).

**Figure 3:**
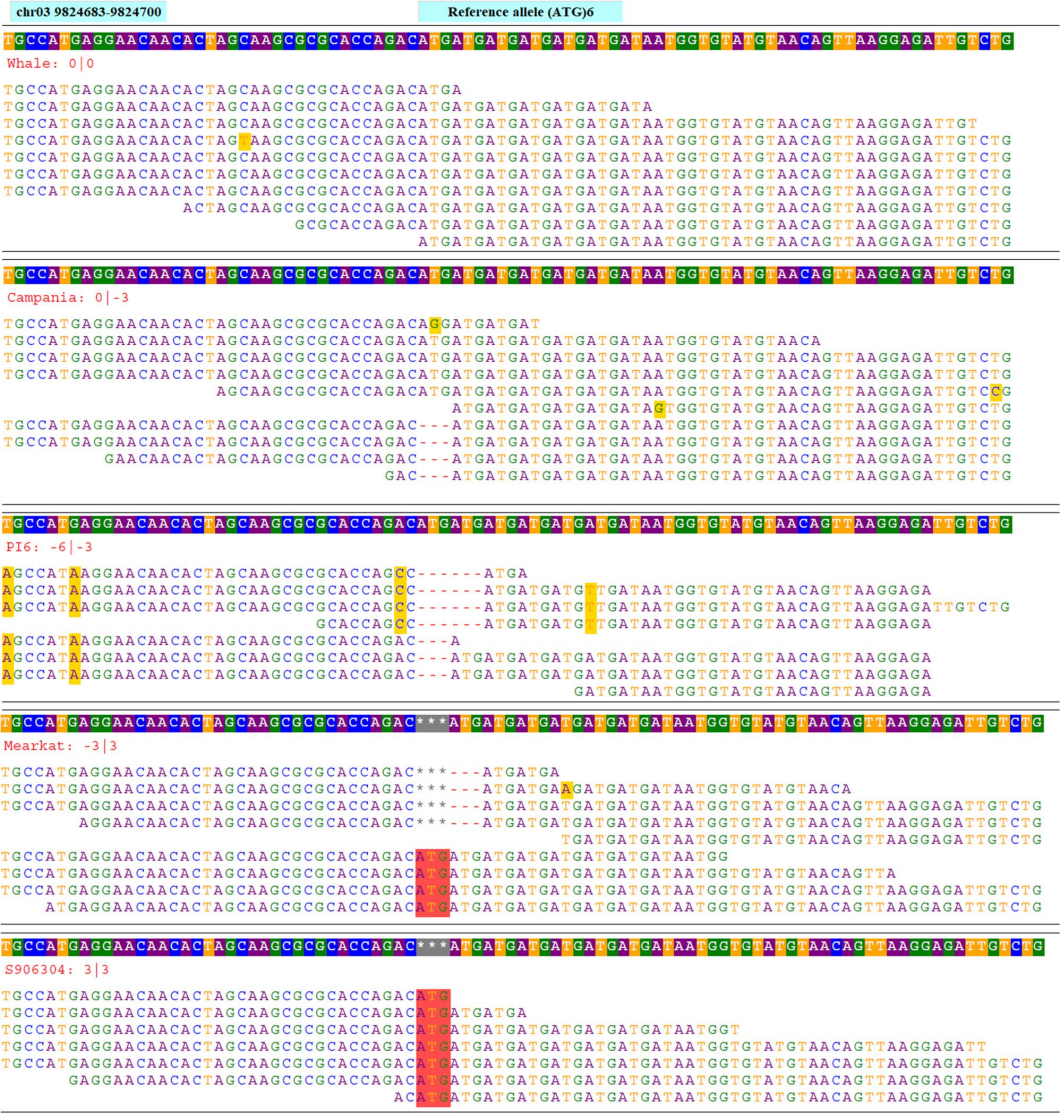
Genome sequences alignments of tri-repeat SSR loci chr03_3_9824683 comprised of 'ATG' motif repeating for six times (ATG)6 and located on chromosome 3 at 9,824,683 bp in the reference genome. Genome sequences of multiple accessions were aligned to the reference genome and variation in the number of repeat units for the SSR repeat motif 'ATG' among the accessions were recorded. The reference sequence containing SSR loci is displayed on the top row while the allele sizes of each accession are displayed on the second row and the aligned reads of each accession are presented on succeeding rows.

**Figure 4:**
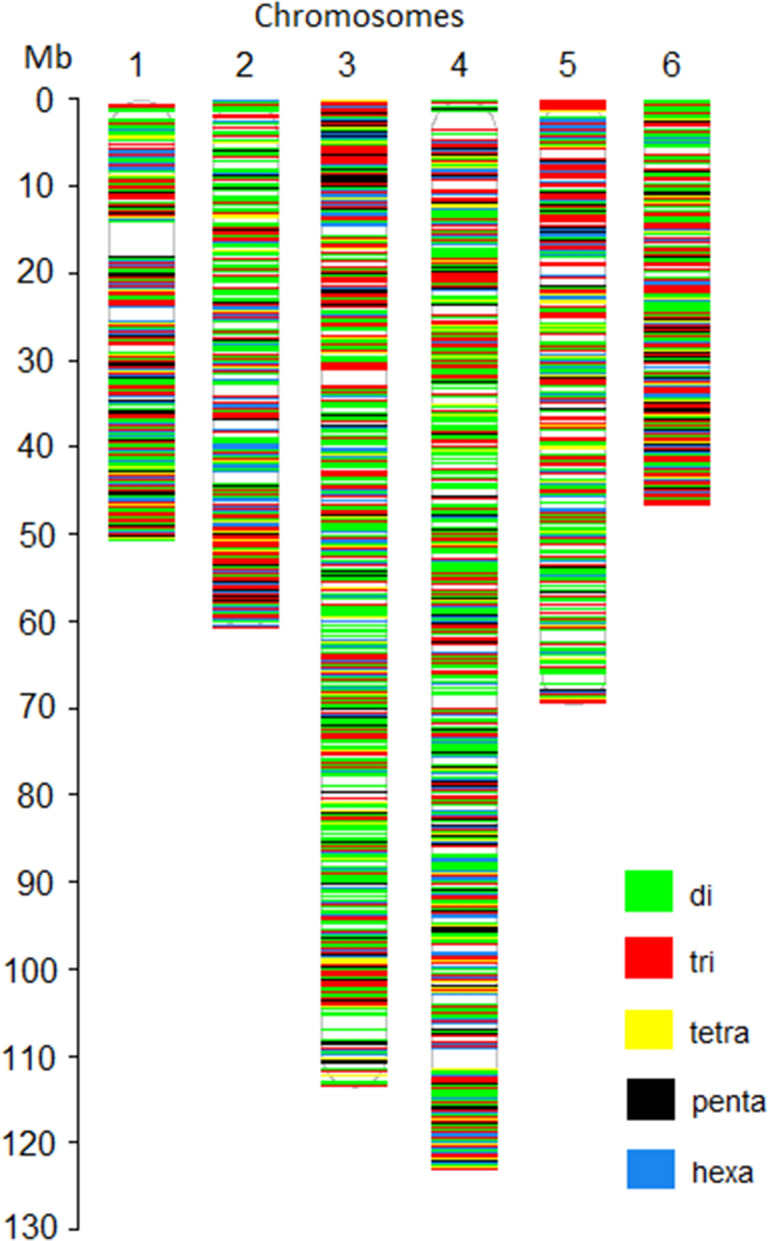
Physical map location and distribution of in silico identified SSR markers from the genome sequence of spinach cultivar Sp75.

Next, a bed format file containing the coordinate of all 19,397 selected SSR loci was prepared. The whole-genome sequences of 21 spinach accessions were mapped to the chromosome sequences of the reference genome, and aligned bam files were generated for each spinach accession. Using the HipSTR program, an in silico genotyping was performed using the aligned bam files of 21 spinach accessions, bed files containing coordinates of 19,397 SSR loci, and reference chromosome sequences. The HipSTR program generated genotype calls for 16,197 SSR loci (Table 2). Of these, 8301 loci were either monomorphic or had missing calls in all accessions and were not pursued. Furthermore, 1892 SSR loci were discarded as these contain non-reference alleles on less than two accessions. The remaining 5986 SSRs showed non-reference alleles in more than two accessions among the genome sequences of 21 spinach accessions that were retained as the set of polymorphic SSR loci. The HipSTR alignment of the genome sequences, in silico genotyping, and allele calling approach used in this study is shown in Fig. 3 for tri-repeat SSR loci (ATG)6 located on chromosome 3 at 9,824,683 bp of the reference spinach genome. The physical map showing the distribution of the 5986 polymorphic SSR loci in the spinach genome was drawn using unique colors for each SSR motif length (Fig. 4). Detailed information of these in silico identified 5986 polymorphic SSR loci is provided in the Supplementary Table S2, which includes a physical position on the reference genome, reference SSR motifs, repeat numbers, and primer pairs for PCR amplification. In addition, 250 bp flanking sequences on both sides of the SSR loci and the number of alleles identified following in silico genotyping in the set of 21 spinach accessions is provided (Supplementary Table S2). Of the polymorphic loci identified in this study, the di- and tri-repeat loci were more abundant, while the other (tetra, penta, and hexa) were relatively lower in number (Table 2). Chromosomes 3 and 4 were the longest and harbored more SSRs, and as expected, they contain a higher number of polymorphic SSRs. The polymorphic SSRs identified here were evenly distributed throughout the six chromosomes, although some regions had higher densities and gaps (Fig. 4, Table 2). A search of the physical location of all polymorphic SSRs for an overlap with the genes on the reference genome GFF files found 36.54% of the total polymorphic SSRs were located on genic regions, and the gene name and their predicted functions for these genic SSRs were reported (Supplementary Table S3).

### Molecular validation and characterization

Thirty-six SSR loci were randomly selected from the set of in silico identified polymorphic SSR loci and genotyped using molecular assay on a panel of 48 spinach accessions to confirm and validate the polymorphism potential of the in silico identified SSR loci (Table 3). The 36 SSRs were distributed evenly across all six spinach chromosomes, having 1 di, 23 tri, 4 tetra, 2 penta, and 6 hexanucleotide repeats. Primers were redesigned for some loci to fit multiplexing during molecular validation. Of the 36 SSR loci, 34 loci gave clear polymorphic band profiles among the spinach panel in this study, while two primer pairs did not amplify (Table 3). The amplification success rate of the primer pairs used to amplify in these accessions set was 94.4%. Importantly, all 34 polymorphic loci selected following computational genotyping achieved 100% polymorphism upon molecular validation assay in a panel of 48 *Spinacia* accessions. A total of 101 alleles were scored at these 34 SSR loci ranging from 2 to 5 alleles per locus and included an average of 2.9 alleles per locus (Table 3). Expected heterozygosity or gene diversity (H_e_) ranged from 0.24 to 0.49 with an average of 0.40 per locus. The polymorphic information content (PIC) of SSR markers ranged from 0.09 to 0.35 and averaged 0.25 (Table 3).

**Table 3:** Repeat motifs, primer sequences, annealing temperatures, and characteristics of 36 simple sequence repeat (SSR) markers in the 48 diverse spinach accessions.

SN	Marker name^a^	Repeat motif	Forward primer^b^	Reverse primer	Product size	Tm (°C)	Na	H_e_	PIC
1	chr01_3_3996646^a^	(ACA)11	CTACAATGGAACCACCCTAGC	TAGTTCAGGCTATCAGCTCCAA	114	59	4	0.42	0.31
2	chr05_3_22933154	(ACA)23	TCCTCTCTCCATTTGCAACC	CCCCAACTCTTCTTGTCAGC	173	60	5	0.41	0.30
3	chr03_4_53804930^a^	(TTGT)7	CGAGCTGTGAAGGTATTTCTTG	GAATTTGTGTTTTGCGCTTC	213	59	2	0.49	0.25
4	chr04_5_76909578	(CAGGC)4	TTCCGGTGTTCTGAAACTACA	TTCCTTTCATTGTTGCTTGG	272	59	–	–	–
5	chr06_6_22506385	(AGGCAA)6	ACAGCACAAGCCACAAAATG	GTGGTACTGGCGGTAGTGCT	307	60	3	0.33	0.28
6	chr04_3_112691959	(ACA)13	AACAAATTCGCACCACATCA	AAGATGTTGGCCGACGATAG	399	60	3	0.38	0.27
7	chr03_3_99679126^a^	(TTG)10	GAAAACTTCGCGTTACAATGAG	CATTTCCGAGTGATCTTGAGC	122	59	3	0.45	0.32
8	chr06_2_37161425	(GA)8	GAGCTTAACAACCGAGCTTGA	GGAATTTTGGGTTGACGTGT	185	60	2	0.48	0.24
9	chr06_3_40415854^a^	(TCA)10	AGCCTATACACACTCCTCTCGG	CTTCTAGGTCCGGTTTCCATC	221	60	3	0.26	0.22
10	chr02_3_30545229	(AGA)10	ATCCCAGCAGTAAACCAACC	AACAGCTTCCTTGCTTGCAT	292	59	3	0.39	0.27
11	chr04_3_54423372	(ACC)5	AGAAACAAAGGCGAGGTACG	CAGATATCGGAATCGCAAGA	340	59	2	0.44	0.18
12	chr02_3_41041559	(TCA)13	GGCTCACCATTTTGGTCATT	ATTGGAAGCATGCAAGGAGT	389	60	3	0.47	0.27
13	chr02_5_18463938^a^	(AGGGA)4	TTAGCAAACACCAAAAATTGGA	GAACACCAAATGTTGCATGG	126	60	2	0.24	0.12
14	chr02_6_52639756	(AGAGAA)4	AAACCACATACAGGGCCTCA	TACACCACACAGCGTCCACT	187	60	3	0.47	0.30
15	chr05_3_55104642^a^	(GAG)6	TTAAGTCAGAAACCTTCCCCTG	TCTTCCATCATTTACGCCAAG	225	60	2	0.46	0.21
16	chr03_6_29450333	(CAAATT)4	ACCCAATAAAACAGCGATGC	CCAAGCTGTCACAAAACACG	276	60	2	0.45	0.24
17	chr02_3_60571150	(AAC)11	AGCTAAACTCCGTGGCACAT	AGAAGGGAGTGGAGGGAGAG	313	60	3	0.44	0.23
18	chr05_3_32205256	(ATC)9	ATGTTGGCCTGATCCATTGT	CGTGAGCAACTGATGGGAAT	388	61	2	0.34	0.22
19	chr03_3_9824683^a^	(ATG)6	CCATGAGGAACAACACTAGCAA	CCATCTCACAGACAATCTCACAG	108	60	2	0.36	0.13
20	chr01_3_47220474	(TTG)7	GGTTCATGTAGCCGACCCTA	AGGTGGAGAACAAGGAAAGAA	189	59	3	0.36	0.27
21	chr06_3_17100717^a^	(TCT)7	TGATACCAATACCTGCACACAA	GGTGTTAAAGGACCCGTTTTC	241	59	2	0.41	0.18
22	chr04_3_42840013	(GCA)9	AAGCTTCCATCTTTGGCTCA	AAAGTCGCTTGCAGTCGTTT	293	60	–	–	–
23	chr03_3_75066435	(AGA)17	CTTGCCTCCAATTTGCATTT	TGTGAGAGTCGGAGAATCCTT	339	59	2	0.41	0.30
24	chr03_3_20380054^a^	(TTC)11	CCTACCACTTTCATCTCATCCA	GTGTAAACAGAACCACCCATGA	391	59	3	0.42	0.32
25	chr05_3_1063291^a^	(TCA)17	ATGAAGGCAAGATTTCGGTACT	CTCAACCACGACATGAACCTTA	135	60	4	0.36	0.23
26	chr04_3_31208653^a^	(CAG)10	CCTCATCATTGTCCTCATCAAG	ACTGCCGAGAACTGCATAAACT	173	60	4	0.46	0.32
27	chr03_3_295631^a^	(GAA)9	CAAAATGGAAACCCCAACAA	CACAGAACAACCCAAACTCAAA	216	60	3	0.36	0.27
28	chr05_4_64332729^a^	(TGTA)9	GGTTTCATCAATCTTCCTGCAT	CCAGGTTCAAAATGCTCCATA	257	60	5	0.41	0.35
29	chr06_3_44057430	(CCA)6	CCACAAACTGCCCTAAATGG	AACCATGGCTCATGTTGATG	304	60	2	0.39	0.14
30	chr01_4_18090562^a^	(TATC)6	AAAGGGCAGAGAGAAGAAAGATG	TCATGCTGAAGTGACCGAAC	350	60	2	0.35	0.09
31	chr01_6_26485152^a^	(CAACAG)4	AACCAAAAGGCAACAAAAATG	TTTTGAACAACCCCGATAGC	135	59	4	0.42	0.29
32	chr05_6_42561823^a^	(CTGCTA)5	TGGTGGCTGGTCATAAGTATTG	CTAACAGAAGCAGCAGCAGAAG	175	60	3	0.40	0.26
33	chr01_6_42933346^a^	(GGTGTG)5	GTTGAAGACGCTGTAGTTCGG	CCCCTTCGCAATACTCCTTAAT	238	60	4	0.40	0.31
34	chr01_4_37404206^a^	(ATGT)8	CCAATGCAAGGGTAACACAAT	TCTCAGCCATCTGTTGATCTGTA	290	60	3	0.35	0.24
35	chr04_3_5365603	(TTC)9	ATCTCGTGCCAACCATAACC	TTGATCAGAGGACAAAGAAAGC	245	59	4	0.40	0.35
36	chr06_3_4451765^a^	(AGG)7	ACCCCATTTCTTCCCTCTTTT	TTAAGATCAACCAACGCCTTCT	380	60	4	0.34	0.29
	Mean						2.97	0.40	0.25

*Na* number of alleles, *He* expected heterozygosity calculated in GenAlEx 6.5, *PIC* polymorphism information content calculated in PowerMarker v3.25.

^a^Primers were redeisgend for these SSR markers.

^b^The M13 (CACGACGTTGTAAAACGAC) tail sequences at the 5′ end of each primers were added, and M13 tailed forward primers used used.

### Phylogenetic and population structure analysis

**Figure 5:**
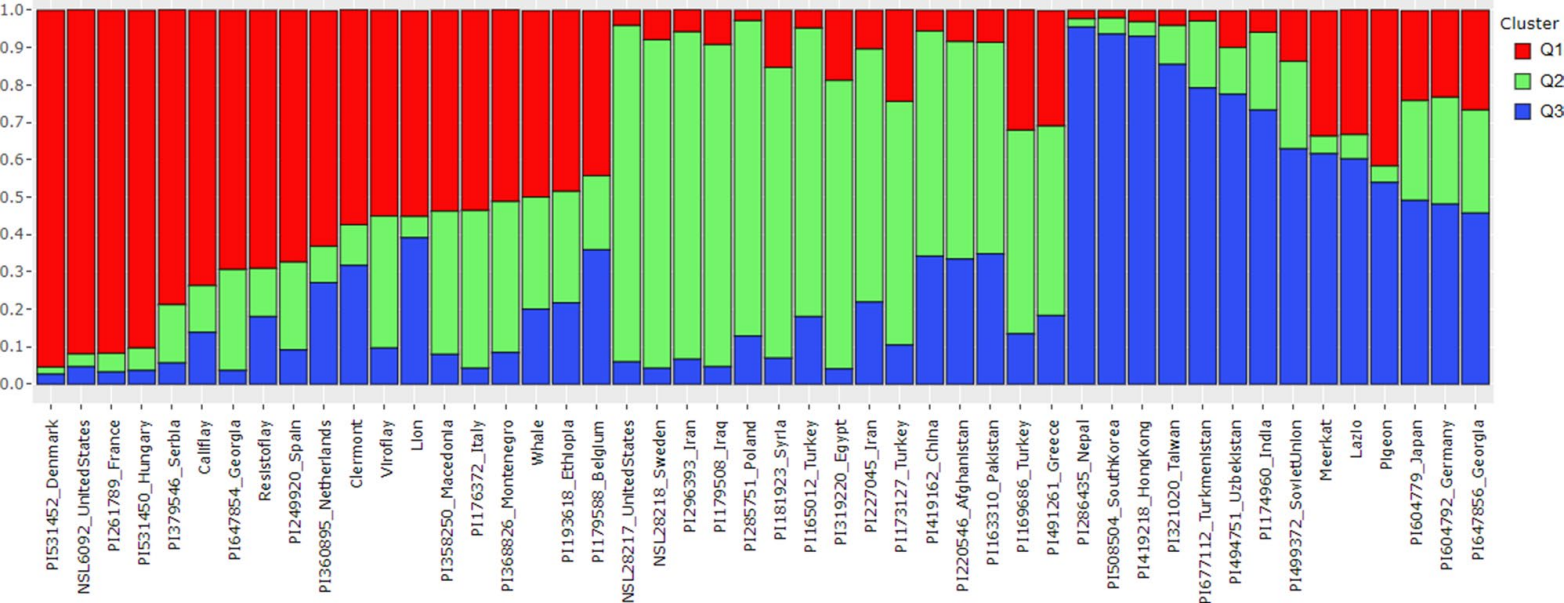
Population structure analysis classified 48 spinach accessions into three population groups based on delta K analysis. The distribution of spinach accessions to the three population clusters Q1, Q2, and Q3 was colored red, green, and blue. The accession name and country of origin are denoted on the x-axis, while the y-axis represents the membership proportion of accession to different population groups.

The STRUCTURE software v.2.3.4^41^ was used to infer the population structure of the 48 spinach accessions for K = 1–10. The Structure Harvester program was used to determine the most likely number of subpopulations that generated the highest delta K value for K = 3 and the second highest was for K = 4 (Supplementary Fig. S1), suggesting the spinach accessions used in this study underlies genetic differentiation of three main populations. We considered reporting the three population groups to assign the spinach accessions genotyped with the SSR markers in this study. A membership probability cutoff (Q value) of 0.50 was used to divide the spinach panel into three (Q1, Q2, and Q3) main populations, while the remaining accessions showing membership proportions Q < 0.5 were grouped as an admixed group (Qm). The classification of spinach accessions into the population groups are provided in Supplementary Table S1. A red, green, and blue color were labeled for the three subpopulations membership proportion Q1, Q2, and Q3 in the STRUCTURE-plot (Fig. 5). The Q1 group comprised 17 accessions (35.4%), mostly the commercial spinach cultivars and landraces from Europe and the USA. Fifteen accessions (31.3%) were clustered in the Q2 group that comprised *S. oleracea* accessions from Central and Western Asia (Iran, Iraq, Turkey, and Syria). Accessions from China, Afghanistan, Pakistan, and Greece merge in the Q2 population, although these accessions also contain a significant ancestry proportion from the Q3 population. The Q3 group comprises 11 accessions (22.9%), including *S. oleracea* landraces from South and Eastern Asian countries (Nepal, South Korea, Hong Kong, Taiwan, and India). The *S. turkestanica* accessions from Turkmenistan and Uzbekistan and *S. oleracea* accession from the former Soviet Union also lie in the Q3 group along with the South and East Asian accessions. In addition, a few *S. oleracea* commercial cultivars (Meerkat, Lazio, and Pigeon) were also grouped in the Q3 population but shared 33–42% ancestry proportion to Q1 subpopulations. The remaining five accessions (10.4%) fell in the admixed Qm group. Of the admixed population, *S. turkestanica* accession from Germany and the *S. oleracea* accessions from Japan and Georgia fell in the admixed group but comprised more than 45% ancestry proportions to the Q3 subpopulation and were merged with the other Q3 accessions. On the other hand, the accessions from Ethiopia and Belgium showed a high ancestry proportion (48 and 44%) to the Q1 group.

**Figure 6:**
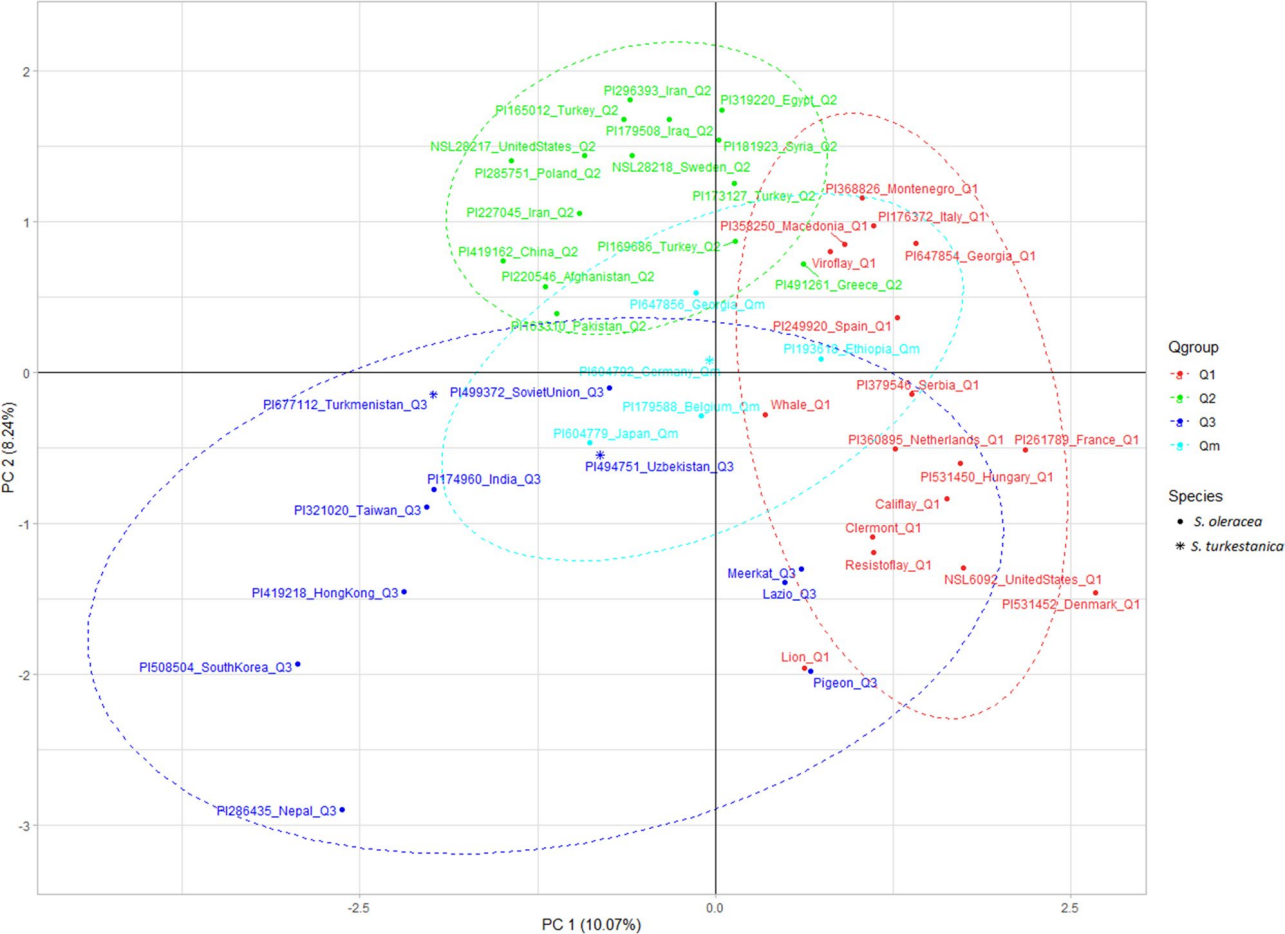
The principal component analysis (PCA) plot for 48 spinach accessions using 34 SSR marker data. The same color code was used for accessions belonging to Q1, Q2, and Q3, and cyan color for the admixed (Qm) population. The *S. oleracea* and *S. turkestanica* species are designated with circle and star shapes.

The genetic divergence among the accessions was further explored by performing PCA analysis in TASSEL 5.2.65^44^. The spinach accessions differentiated into three main clusters in the PCA plot (Fig. 6). Spinach accessions assignment in the PCA corresponded well to the STRUCTURE assigned Q groups with some mismatches and overlapping. The accessions were grouped into three major populations and were colored using the same color code as in the STRUCTURE plot, while cyan color was used for the admixed group (Qm). The first two principal components, PC1, and PC2 explained 10.07% and 8.24% of the overall molecular variance, and the two PC separated the accessions into three population groups, despite some overlaps. Cluster 1 contains the Q1 accessions, cluster 2 contains Q2 accessions, cluster 3 contains Q3 accessions, and the admixed accessions are colored with cyan color are in the center of the three clusters.

**Figure 7:**
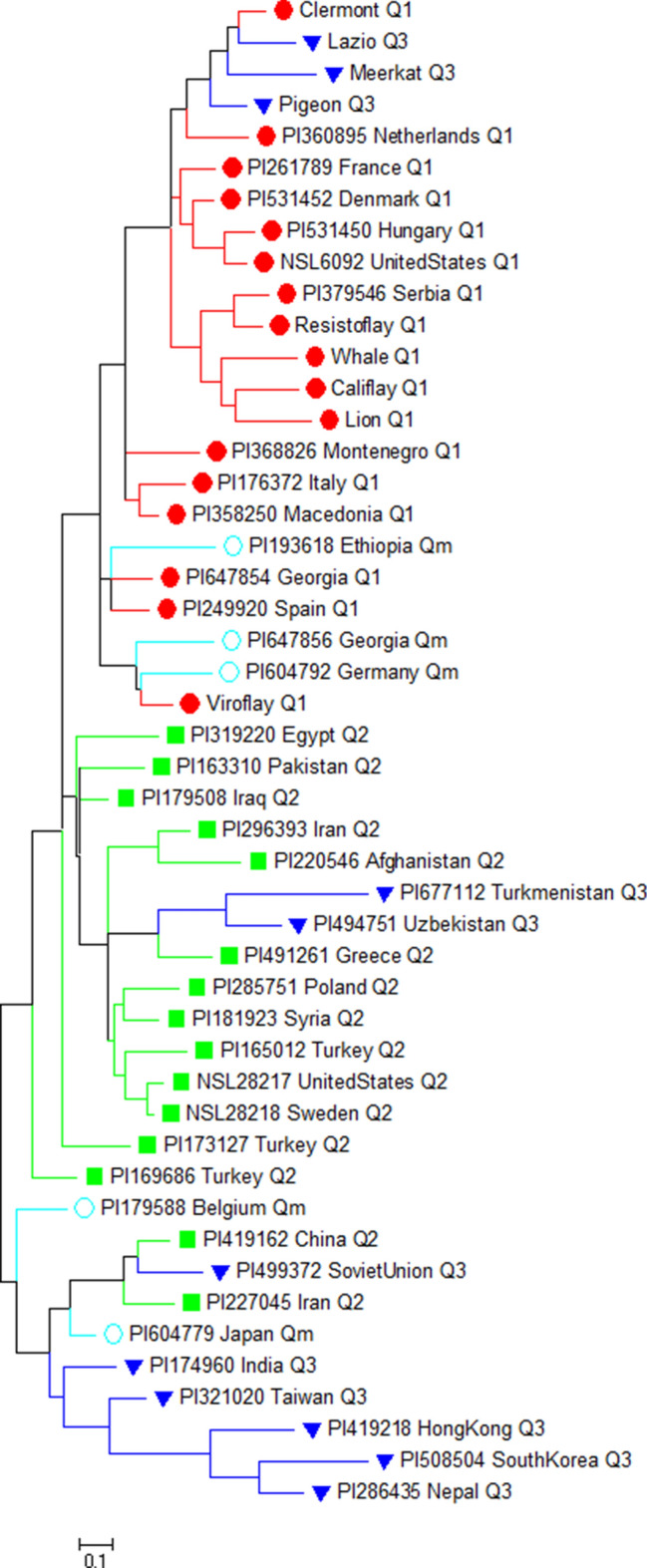
The maximum likelihood Neighbor-joining tree of 48 spinach accessions drawn in MEGA. The accessions color codes are consistent with the PCA and STRUCTURE-plot.

The genetic diversity in the spinach panel was analyzed using the maximum likelihood method based on the Tamura–Nei model^45^ in MEGA 7^46^. The phylogenetic analysis of 48 spinach accessions with the newly developed genome-wide 34 SSR markers showed three separate spinach accession clusters with some overlaps (Fig. 7). The same set of red, green, blue, and cyan colors was used to indicate Q1, Q2, Q3, and Qm groups as used for accessions in the MEGA and PCA analysis. The accessions from South and East Asia (Q3 group) form a separate cluster, close to the West Asian accessions, and were distant from the European and United States accessions and the differential cultivars. The *S. turkestanica* accessions from Turkmenistan and Uzbekistan grouped with the accession from West Asian countries Greece, Syria, Turkey, Afghanistan, and Iran in the Q2 cluster, alough these two accessions were grouped closer with the Q3 accessions in STRUCTURE and PCA plots. In contrast, the *S. turkestanica* accession from Germany grouped with accession from Georgia and differential cultivar Viroflay in the Q1 cluster, and this accessions was in the admixed group in all three analysis.

Overall, the diversity analysis indicates three well-differentiated populations in the worldwide spinach panel, and the accessions were consistent in the neighbor-joining tree, STRUCTURE, and PCA results despite some overlaps and mismatches (Figs. 5, 6, 7). Compared to the STRUCTURE result, the accessions were more clearly visualized and differentiated in the NJ tree and PCA plots. Genetic grouping of spinach accessions based on SSR markers corresponded well with their geographical origin, domestication history, and pedigree. And the genetic clustering of accessions was similar to previous genetic diversity studies^26,47^.

## Discussion

The advancement of sequencing technologies and the availability of genome sequences for many commercial and specialty crops have eased the discovery of SSR markers in the previous decade. Previously SSR markers were discovered from the sequencing of genomic libraries enriched for SSRs and were not efficient in terms of cost, time, and technical resources^8,48,49^. The availability of genomic and transcriptomic sequences has increased the rate and efficiency of developing SSR markers, and SSRs have been developed in the model, non-model, and orphan crops^50–52^. However, many of the recent SSR development studies, particularly using the whole-genome and transcriptome sequences, tested a small number (generally between 20 and 100) of randomly selected SSR loci to report a few useful polymorphic markers. The challenge remained in screening and identifying polymorphic loci with variable repeat length across the panel. Here, we present a new approach to discovering polymorphic SSR markers by aligning and comparing the genome sequences of multiple accessions against the reference genome to count and genotype SSR loci variation in repeat length. A subset of computationally identified polymorphic SSR loci using the HipSTR program^32^ was validated in a set of 48 diverse spinach germplasm accessions to confirm polymorphism in PCR based molecular assay. The availability of reference genome of spinach and the genome sequences of several accessions with an average depth of 30× have enabled us to discover thousands of polymorphic loci rapidly. This study efficiently discovered a large set of genome-wide SSR markers with known physical map locations across the six spinach chromosome.

The SSR distribution in the Sp75 genome was previously reported^20^ and is not described in detail here. This study aimed to generate a large set of polymorphic SSR loci to extend their use in future genetic studies in spinach. Initially, primer pairs were only designed for 35,567 loci, while primers were not designed for 6588 SSR loci due to lack of flanking sequences and missing sequences in the template sequences to design primers (Table 2). The mononucleotides are error-prone in scoring and were not pursued in this study, while the dinucleotide repeats are high in stuttering^53,54^. In contrast, the tri and higher nucleotide containing motif are easy to score as are less prone to amplification errors and stuttering, although the higher repeat SSRs were present in a low frequency in spinach. Hence, the SSRs with higher repeat numbers (tri, tetra, penta, and hexa) are recommended for future genetic studies.

Forty-eight spinach accessions from two *Spinacia* species, *S. oleracea* and *S. turkestanica,* were used to confirm the polymorphism potential of the computationally identified polymorphic SSR markers using the molecular genotyping assay. The two primer pairs do not amplify in the spinach panel and most likely because of the addition of the M16 tail that interfered with amplification by producing secondary structures and changes in annealing temperatures. Genotype results from the molecular assay for the remaining SSR loci completely corresponded to the computation generated genotype profile as all 34 markers showed two or more bands across the panel. Identification of 100% of polymorphic loci from a random test of 34 computationally screened SSR loci is a promising approach to identify polymorphic SSR loci for the organism with available genome sequences. The result indicates most of the in silico derived SSRs reported in this study are truly polymorphic. Primer sequences were designed for all 5986 SSR loci, and flanking sequences for all loci have been provided (Supplementary Table S2). The flanking sequences can be used to redesign primers with different product sizes to fit in the multiplex runs. The two primer pairs do not amplify in this study but could be amplified with different sets of primers but were not tested here. The majority of the polymorphic SSRs reported here were positioned in the intergenic regions, with 36.54% distributed in the gene regions (Supplementary Table S3) even though a lower proportion of SSRs are known to reside in the gene region, as reported in other organisms^55,56^. Comparable to our report, 29–30% of the SSR markers developed from the genome sequences in carrot and hazelnut belong to the transcribed regions^12,57^. Also, the tri and hexa repeat loci have been more prevalent in the transcribed regions, and those two motifs comprise 49% of total SSRs in this study, apparently leading to high genic SSRs. The high percentage of genic markers plus the gene and gene functions reported here may help in breeding and physiological studies. A large set of SSR markers identified in this study will support genetic, genomic studies in spinach, mainly in fingerprinting, genetic diversity analysis, marker-trait linkage and association analysis, and molecular breeding. This approach of discovering polymorphic SSR markers is resource-efficient both in terms of time and cost. On top of that, the method reported in this study is transferable to other species with available genome sequences and resequences data.

The relatively lower number of alleles (range of 2–5 with an average of 2.9 alleles/locus) reported in this study is due to a careful and strict allele scoring employed here. The average PIC value of the markers in this study was 0.25, which is slightly lower than the previous report in spinach (average PIC 0.43)^20^ and is explained by the low number of alleles reported in this study. The use of fluorescently labeled primers in the multiplex set and fragment analysis in capillary electrophoresis can increase allele calling at high precision with clearer results and eliminate the difficulties with scoring bands in the agarose and polyacrylamide gel electrophoresis method. Hence, we expect and recommend using semi-throughput capillary electrophoresis methods for fragment sizing to generate clearer allele sizes and record a higher number of alleles. The number of alleles genotyped by the HipSTR program among the genome sequences of 21 accessions and the reference genome is provided (Supplementary Table S2), giving us an idea of the expected number of alleles while designing future experiments to use these markers.

Fingerprint data generated from PCR genotyping was used to assess the genetic diversity and population structure of the spinach accessions. The population structure and phylogeny assignment performed with allele scores of 34 SSR markers in this study distinguished the worldwide spinach germplasm accessions and the population group assignments were consistent with previous reports^26,47^. The accessions were subdivided into three main populations based on STRUCTURE analysis, and the phylogenetic analysis in MEGA and PCA analysis in TASSEL supports the three genetic clusters. The clusters generated by structure analysis, neighbor-joining analysis, and PCA were similar. The accessions assigned to the population group largely correspond to the geographical origin for most accessions, with a few mismatches and overlaps. Few differential cultivars (Meerkat, Lazio, and Pigeon) were assigned to the Q3 group along with the Southern and East Asian accessions in Structure analysis. However, the differentials were merged with the Q1 group in PCA and phylogenetic analysis. Germplasm from Pakistan and Afghanistan were grouped with the Q2 population (Central and Western Asia) further away from India and Nepal (Q3 population). But a recent diversity analysis study^26^ generated similar results where the South Asian accessions formed multiple clusters, and some were close to accessions from Western Asia. The phylogenetic trees clustered the two *S. turkestanica* accession from Turkmenistan and Uzbekistan together with the *S. oleracea* accessions from South and West Asian countries Afghanistan, Pakistan, Greece, and Turkey. The *S. tetrandra* and *S. turkestanica* are the wild relatives of cultivated spinach *S. oleracea*. Previous studies reported *S. turkestanica* was genetically close to cultivated *S. oleracea*^26,27^. Successful amplification of accessions belonging to two *Spinacia* species (*S. turkestanica* and *S. oleracea*) by the same primers and the accessions from two species lying together in the phylogenetic analysis in this study further supports the hypothesis that *S. turkestanica* is the recent ancestor of *S. oleracea*.

Efforts to identify genes for major traits are prioritized in spinach. Regardless of the reduced cost of whole-genome sequencing and reduced representation sequencing (GBS and RADseq), genotyping small sets of SSR markers are economical and easy to manage^58^ and are helpful to make the framework genetic studies. Downy mildew is the most important disease that devastated all major spinach production areas, specifically the California valleys, the major spinach production area in the United States. Previous genetic mapping efforts have attempted to map the trait locus but have been limited with the availability of a dense set of molecular markers resulting in a lower resolution of the trait locus. The availability of physically mapped markers can help expedite fine-mapping research and studying the genetic control of the trait at a finer resolution. Abundant SSRs identified in this study may be used in the beginning to map the targeted trait locus via association or QTL mapping, followed by SNP markers to narrow the locus interval. For fine mapping and in-depth analysis, targeted sequencing methods as amplicon sequencing^59,60^ and hybridization-target enrichment^61,62^ can be employed to generate sequence data of targeted regions at high coverage to gain insights on the genetic basis of trait control. Markers including SSRs in the proximal end of spinach chromosome 3 are promising as the known downy mildew resistance locus (known as RPF) maps in the region. The RPF loci have been mapped to the 0.3–1.3 Mb region of chromosome 3^63–66^. These new SSR markers may also help develop near isogenic lines (NIL) to track the resistance gene introgressed region of the recurrent susceptible lines. Furthermore, the genome of some races of spinach downy mildew pathogen (*Peronospora effusa* race 1, 12, 13, 14) has been sequenced^67–69^. These sequences could be searched to identify the set of SSRs varying among the races, identify a genome-wide fingerprint and diagnostic sets of SSR loci, and identify SSRs involved on and/or associated with virulence-pathogenic loci. Such SSR panels could be used routinely in functional diversity analysis and marker profile highly variant and continually emerging new pathogen races. The same approach could be extended for other diseases in spinach and examine and understand host–pathogen interactions in other crops. Furthermore, a new genome sequence assembly (https://phytozome-next.jgi.doe.gov/info/Soleracea_Spov3) is available in spinach, and additional 480 USDA accessions, breeding lines, and commercial cultivars are resequenced at 10× genome coverage^70^. These new sequences generated for spinach will facilitate the identification of more SSR loci and map them across longer chromosome lengths.

## Conclusions

This study utilized the available reference spinach genome sequences to mine SSR loci along with the genome sequences of additional accessions to develop a large set of polymorphic SSR markers following computational screening for the variation in the number of repeat units among the accessions. Substantiate polymorphism observed following molecular validation of randomly selected SSR loci demonstrated our strategy to identify new polymorphic SSR markers. The development of a large set of polymorphic SSR markers in this study will support genetic research in spinach, especially for the labs with limited resources. A dense set of relatively easy and inexpensive to use SSR markers reported in this study may facilitate fingerprinting, genetic diversity, phylogeny assignment, population structure analysis, and mapping and molecular breeding effort in spinach. Notably, the polymorphic SSR markers can be employed to investigate genetic diversity and population structure among the wild and cultivated spinach accessions and to identify duplicates and generate a core set of diverse accessions. Indeed, these markers can be equally valuable for breeding applications to investigate and map the major and minor traits. Most importantly, our approach of identifying polymorphic SSRs will expedite the development of useful markers with the known physical location and avoids laborious preliminary molecular screening for polymorphism.

## Supplementary Information

Supplementary Information 1.

Supplementary Information 2.

## Data Availability

Data generated in this study are available in the main table, figures, and additional files, and the raw sequencing data are available at the National Center for Biotechnology Information SRA database under the accession PRJNA860974.
