# Author Correction: Genome-wide simple sequence repeats (SSR) markers discovered from whole-genome sequence comparisons of multiple spinach accessions

**DOI:** 10.1038/s41598-022-18803-7

**Published:** 2022-08-23

**Authors:** Gehendra Bhattarai, Ainong Shi, Devi R. Kandel, Nora Solís-Gracia, Jorge Alberto da Silva, Carlos A. Avila

**Affiliations:** 1 Department of Horticulture, University of Arkansas, Fayetteville, AR 72701 USA; 2 Texas A&M AgriLife Research and Extension Center, Weslaco, TX 78596 USA; 3 Department of Crop and Soil Sciences, Texas A&M University, College Station, TX 77843 USA; 4 Department of Horticultural Sciences, Texas A&M University, College Station, TX 77843 USA

Correction to: *Scientific Reports* https://doi.org/10.1038/s41598-021-89473-0, published online 11 May 2021

The original version of this Article contained an error.

The accession codes for the raw sequencing data generated in the course of the study were omitted from the Data Availability statement.

“Data generated in this study are available in the main table, figures, and additional files.”

now reads:

“Data generated in this study are available in the main table, figures, and additional files, and the raw sequencing data are available at the National Center for Biotechnology Information SRA database under the accession PRJNA860974.”

The original Article has been corrected.

