# Supplementary material for: Genome-wide simple sequence repeats (SSR) markers discovered from whole-genome sequence comparisons of multiple spinach accessions
Source: Sci Rep. 2021 May 11;11:9999. doi: 10.1038/s41598-021-89473-0 (PMC8113571; doi:10.1038/s41598-021-89473-0)
Supplement: Supplementary file 1 — Supplementary Information 1. [file 41598_2021_89473_MOESM1_ESM.docx]

Genome-wide simple sequence repeats (SSR) markers discovered from whole-genome sequence comparisons of multiple spinach accessions

Gehendra Bhattarai^1^, Ainong Shi^1*^, Devi R. Kandel^2^, Nora Solís-Gracia^2^, Jorge Alberto da Silva^2,3^, Carlos A. Avila^2,4*^

^1^Department of Horticulture, University of Arkansas, Fayetteville, AR 72701, USA.

^2^Texas A&M AgriLife Research and Extension Center, Weslaco, TX, 78596, USA.

^3^Department of Crop and Soil Sciences, Texas A&M University, College Station, TX, 77843, USA.

^4^Department of Horticultural Sciences, Texas A&M University, College Station, TX, 77843, USA.

*Corresponding authors:

**Ainong Shi**

University of Arkansas.

Fayetteville, AR 72701

Phone: 479-575-2670

Email: [ashi@uark.edu](mailto:ashi@uark.edu)

**Carlos A. Avila**

Texas A&M AgriLife Research and Extension Center

Weslaco, TX 78596

Phone: (956) 969-5636

Email: [Carlos.Avila@ag.tamu.edu](mailto:Carlos.Avila@ag.tamu.edu)


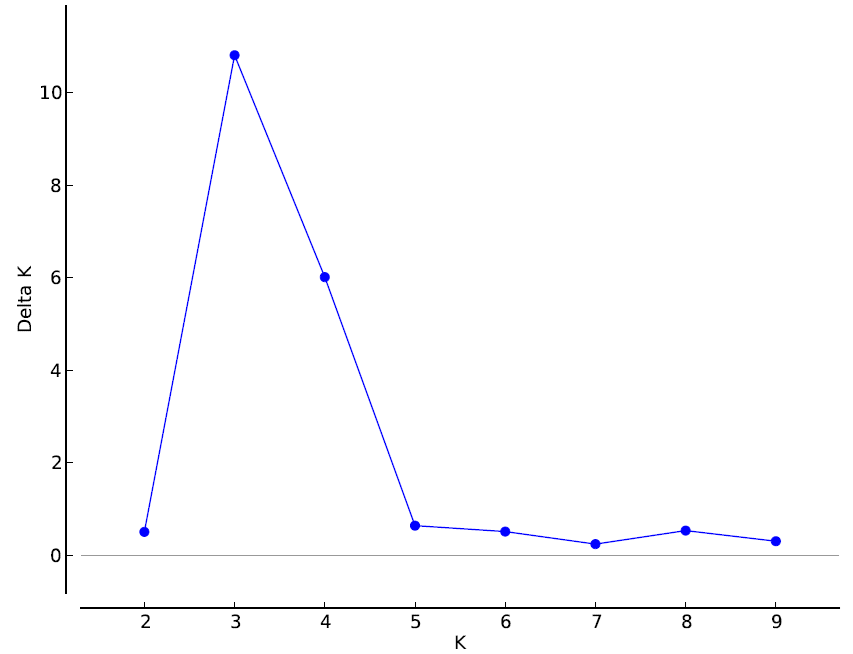


Supplementary Figure S1: Optimal K was determined using the delta K estimation method following STRUCTURE analysis.
